# How parents and practitioners experience research without prior consent (deferred consent) for emergency research involving children with life threatening conditions: a mixed method study

**DOI:** 10.1136/bmjopen-2015-008522

**Published:** 2015-09-16

**Authors:** Kerry Woolfall, Lucy Frith, Carrol Gamble, Ruth Gilbert, Quen Mok, Bridget Young, the CONNECT advisory group

**Affiliations:** 1 Department of Psychological Sciences, University of Liverpool, Liverpool, UK; 2 Department of Health Service Research, University of Liverpool, Liverpool, UK; 3 Department of Biostatistics, University of Liverpool, Liverpool, UK; 4 UCL Institute of Child Health, London, UK; 5 Great Ormond Street Hospital for Children NHS Foundation Trust, Great Ormond Street, London WC1N 3JH, London, UK

**Keywords:** MEDICAL ETHICS, QUALITATIVE RESEARCH

## Abstract

**Objective:**

Alternatives to prospective informed consent to enable children with life-threatening conditions to be entered into trials of emergency treatments are needed. Across Europe, a process called deferred consent has been developed as an alternative. Little is known about the views and experiences of those with first-hand experience of this controversial consent process. To inform how consent is sought for future paediatric critical care trials, we explored the views and experiences of parents and practitioners involved in the CATheter infections in CHildren (CATCH) trial, which allowed for deferred consent in certain circumstances.

**Design:**

Mixed method survey, interview and focus group study.

**Participants:**

275 parents completed a questionnaire; 20 families participated in an interview (18 mothers, 5 fathers). 17 CATCH practitioners participated in one of four focus groups (10 nurses, 3 doctors and 4 clinical trial unit staff).

**Setting:**

12 UK children's hospitals.

**Results:**

Some parents were momentarily shocked or angered to discover that their child had or could have been entered into CATCH without their prior consent. Although these feelings resolved after the reasons why consent needed to be deferred were explained and that the CATCH interventions were already used in clinical care. Prior to seeking deferred consent for the first few times, CATCH practitioners were apprehensive, although their feelings abated with experience of talking to parents about CATCH. Parents reported that their decisions about their child's participation in the trial had been voluntary. However, mistiming the deferred consent discussion had caused distress for some. Practitioners and parents supported the use of deferred consent in CATCH and in future trials of interventions already used in clinical care.

**Conclusions:**

Our study provides evidence to support the use of deferred consent in paediatric emergency medicine; it also indicates the crucial importance of practitioner communication and appropriate timing of deferred consent discussions.

**Strengths and limitations of this study**

- This is the first UK study to explore the views and acceptability of deferred consent among parents and practitioners with first-hand experience of such a consent process.
- Our mixed method interview, survey and focus group study involved 275 parents and 17 practitioners from 12 of 14 CATheter infections in CHildren (CATCH) trial sites. We maximised diversity within our qualitative sample by selecting for interview both mothers and fathers; those who had consented and those who had declined consent for CATCH; as well as parents who did and those who did not have experience of deferred consent.
- Our study provides evidence that can be used by practitioners and patient and public involvement partners involved in the design, ethical approval and conduct of children's critical care trials to improve how consent is sought in the emergency setting.
- Opportunities to purposively sample parents who declined consent and bereaved parents were limited due to high consent rates and low death rates in the trial. This limits our understanding of the experiences of these groups, and the recommendations that we can make to inform how deferred consent should be sought when a child has died.
- Attempts were made to include children in our study; however, their assent was rarely sought in CATCH. Practitioners attributed this to children either being too young or being sedated, and there being a limited window of opportunity for discussions prior to discharge.

## Introduction

Improvements to life-saving treatments for critically ill children have been limited by the ethical and practical challenges involved in seeking consent for clinical trial entry.^1^ The process of seeking informed consent requires time, but this is severely constrained in emergency situations, such as acute resuscitation and critical care,^2^ where even minimal treatment delays are likely to be harmful.^3^ Parents are not always present when a child requires emergency treatment or a mother of a critically ill neonate may be sedated. Children's critical care settings are intensely emotional, and some parents may not wish to be approached about research when their child is critically ill.^4^ Such challenges pose difficulties for doctors and nurses recruiting to clinical trials in ensuring that parental consent has been informed, the participation voluntary and the recruitment process adhered to ethical principles.^5^ The last decade has seen international efforts to find alternatives to prospective informed consent so that vital research can continue to advance evidence-based children's medicine.^1^ In the UK and approximately half of the European Union member states, clinical trials legislation amendments^6–10^ have been introduced to enable children (under 16 years) to be entered into a trial without prior informed consent. UK legislation allows this under the following conditions: “(i) treatment is required urgently; (ii) urgent action is required for the purposes of the trial; (iii) it is not reasonably practicable to obtain consent prospectively; and (iv) an ethics committee has approved the consent procedure.”^9^ In the UK, this process has been called deferred consent, although the term is potentially misleading. This is because the requirement to seek consent to the allocated treatment is essentially ‘waived’ in this situation as doctors and nurses only approach the parent *after* the investigatory treatment has been given. Therefore, permission is for use of information that has already *been* collected and for their child to continue in the trial.^11^ The use of the term deferred consent has led to much discussion recently, leading to a move towards the term ‘research without prior consent’. However, in this paper we will use deferred consent as this was the term used with participants during interviews and surveys conducted in this study.

Conducting research without prospective consent has been subject to much debate as there are concerns it reduces personal choice and so erodes individual autonomy.^12–14^ However, deferred consent has been permitted under certain circumstances because it enables important research to proceed, avoids potentially harmful delays to the treatment of very sick children,^15^ ^16^ and it has been argued, avoids burdening extremely anxious parents. However, there are uncertainties about how to approach deferred consent in a way that is acceptable to children, parents and practitioners, and ethically appropriate. Research is needed to address these uncertainties and inform the design, funding, conduct and ethical review of trials in this setting.^17^ While a few studies have indicated general support for the method,^4^ ^18^ ^19^ such studies have not involved parents with direct experience of deferred consent and therefore, the evidence is limited by its hypothetical nature and lack of tangible insight. An exception is a study of an African emergency trial, FEAST, which incorporated a preliminary and brief verbal assent stage before administration of the investigatory interventions, as well as deferred consent following the intervention.^20^ Staff and parents viewed the verbal assent as a way of protecting the interests of researchers and parents, although the authors questioned the validity of verbal assent due to concerns about parents’ understanding and voluntariness at the height of their child's critical illness.^1^ ^20^ Research is needed to explore the views and experiences of families and practitioners in different contexts, and with these experiences of different methods of deferred consent to ensure that future approaches to consent in children's emergency trials are appropriate to the needs of families and ethically acceptable.

We conducted a mixed method study (CONNECT) using interviews, focus groups and questionnaires to explore the views and experiences of parents and practitioners regarding deferred consent in the CATheter infections in CHildren (CATCH) trial.^21^ CATCH was a three-arm pragmatic randomised controlled trial comparing the effectiveness of heparin-bonded or antibiotic-impregnated central venous catheters (CVC) with standard CVCs for preventing hospital acquired blood stream infection. All three catheters were used in routine clinical practice across the UK. Between March 2010 and November 2012, 1859 children were randomised in CATCH across 14 UK hospitals and one emergency transfer service. CATCH used prospective informed consent for elective surgery admissions and deferred consent for emergency admissions (see table 1). The inclusion of both groups in CONNECT provides a valuable opportunity to compare, within the same trial, the perceptions of parents who had experienced deferred consent with the perceptions of parents who had experienced prospective consent.

**Table 1:** CATCH consent procedures

	CATCH emergency arm	CATCH elective arm
Who sought consent?	CATCH research nurse or Principal Investigator	CATCH research nurse or Principal Investigator
When was consent sought?	Usually within 48 h of admission	Prior to surgery
What was the order of consent, randomisation and CVC insertion?	Randomisation CVC insertion and blood sample if there was a clinical indication of infection Deferred consent	Prospective consent Randomisation CVC insertion and blood sample if there was a clinical indication of infection

CATCH, CATheter infections in Children; CVS, central venous catheter.

## Methods

### Study design

As well as generating evidence on stakeholder views and experience of deferred consent,^4^ ^22^ the Wellcome Trust-funded CONNECT study provided an opportunity to consider this evidence in the light of the bioethical literature and thereby, to inform normative guidance about how consent should be sought for children's clinical trials in emergency situations. Between May 2012 and May 2013, we used semi-structured questionnaire followed by interviews to explore parents’ views and experiences of the CATCH trial recruitment, the consent seeking procedures and decision-making (eg, what influences a parent's decision to consent?), as well as their views on how consent should be sought in future children's emergency trials (eg, when is it an appropriate time to approach parents? Is prospective consent appropriate?). The questionnaire included four items from the decision-making control instrument,^23^ and researcher derived statements to which parents responded using a five-point Likert scale (see table 2). We used focus groups with practitioners (CATCH recruiting doctors, nurses and the trial management team) to explore their views and experiences of recruitment and consent seeking; on trial management issues; and the ethical considerations related to informed consent. We chose a mixed method design,^24^ ^25^ which provided us with different forms of data and insights from multiple participant perspectives to enable a more complete picture of the deferred consent process.^26^

**Table 2:** Parents’ survey responses regarding the CATCH consent process (n=275)

	Strongly agree	Agree	Neither agree nor disagree	Disagree	Strongly disagree	p Value
Statement 1: I was satisfied with the consent process for CATCH						
Emergency	74 (47.4)	71 (45.5)	11 (7.1)	0 (0)	0 (0)	0.09
Elective	68 (58.1)	46 (36.3)	3 (2.6)	0 (0)	0 (0)	
Statement 2: I had enough time to think about whether or not to consent for my child to take part in CATCH						
Emergency	81 (51.6)	65 (41.4)	6 (3.8)	4 (2.5)	1 (0.6	0.98
Elective	58 (49.2)	50 (42.4)	6 (5.1)	3 (5.1)	1 (0.8)	
Statement 3: I made this decision						
Emergency	105 (67.7)	38 (24.5)	10 (6.5)	1 (0.6)	1 (0.6)	0.17
Elective	70 (59.3)	42 (35.6)	3 (2.5)	1 (0.8)	2 (1.7)	
Statement 4: The decision about research was inappropriately influenced by others						
Emergency	1 (0.6)	2 (1.3)	7 (4.5)	33 (21.0)	114 (72.6)	0.75
Elective	0 (0)	2 (1.7)	3 (2.6)	21 (18.3)	89 (77.4)	
Statement 5: I understood the information that I received from the doctor/research nurse about CATCH						
Emergency	94 (59.9)	59 (37.6)	2 (1.3)	1 (0.6)	1 (0.6)	0.74
Elective	67 (56.8)	49 (41.5)	2 (1.7)	0 (0)	0 (0)	
Statement 6: I had enough opportunity for questions about CATCH						
Emergency	91 (58.3)	56 (35.9)	6 (3.8)	2 (1.3)	1 (0.6)	0.87
Elective	68 (57.6)	45 (38.1)	3 (2.5)	2 (1.7)	0 (0)	

Figures are given as n (%); Missing responses: Statement 1:1 emergency and 1 elective; Statement 3:2 emergency; Statement 4:3 elective; Statement 6:1 emergency.

CATCH, CATheter infections in Children.

### Recruitment and sampling to the survey

Practitioners sought consent to participate in CONNECT from parents invited to CATCH, including parents who declined consent to CATCH. Practitioners asked parents who wished to take part in CONNECT to complete the questionnaire in their own time and return it to KW in a stamped addressed envelope. The consent form included a reply slip for parents to indicate if they and their children (aged over 7 years) would like to take part in either a telephone or face-to-face interview. To include parents who had consented to CATCH before CONNECT recruitment began, practitioners posted an invitation letter, CONNECT participant information sheet, questionnaire and interview reply slip to parents who had indicated on their CATCH consent form that they would like to be involved in further research.

### Sampling for parent interviews and practitioner focus groups

To maximise sample diversity,^27^ KW used completed questionnaires and interview reply slips to select parents for interview from each participating trial site so as to include: mothers, fathers, bereaved parents, parents who had experienced either deferred or prospective consent, and those who had consented or refused their child's participation in CATCH. KW contacted parents to arrange the interviews. Sampling continued until no new themes were identified.^27^ For the practitioner focus groups, KW invited practitioners (research nurses and consultant grade doctors) at five CATCH sites; sites were purposively selected to include variation in site level rates of recruitment to CATCH, as well as to explore recruitment and consent issues emerging during the course of data analysis. Two members of the CATCH trial management and two members of the data monitoring team based within the Medicines for Children Clinical Trial Unit (MC CTU) were invited to join a separate focus group.

### Parent interviews and practitioner focus groups

Interview topic guides were developed throughout the study by drawing on themes identified from earlier stages of CONNECT^22^ and review of bioethical literature,^28^ and included questions on: recruitment and consent experience; decision-making and motives for participation; child's assent; and future approaches to consent in emergency trials (see online supplementary file 1 for an example interview topic guide). KW conducted all semistructured interviews and focus group discussions. KW explained to parents and practitioners that CONNECT was independent of CATCH. These were orientated around our topic guides to ensure exploration of core topics, yet conversational to ensure the content reflected participants’ own priorities and perspectives. Respondent validation involved continuously updating the topic guides so that topics which participants raised in earlier interviews and free text questionnaire responses could be explored in later interviews.^29^ All interviews and focus groups were audio recorded, transcribed, checked and anonymised.

### Analysis

KW (a sociologist) led the analysis and development of coding framework with assistance from BY (a psychologist) and LF (a bioethicist) to enable investigator triangulation.^27^ ^30^ Analysis was broadly interpretive, exploring parents’ views and accounts of what happened to them so as to clearly understand their meaning, and iterative by referring back and forth between the developing analysis and new data for evidence of parents’ experiences of approaches to recruitment and consent.^31^ ^32^ Themes were, therefore, inductively derived from the data. Analysis was informed by the constant comparison approach with the aim of achieving catalytic validity, whereby findings help to inform future research and practice.^31^ ^33^ We used NVivo to assist in the organisation and indexing of responses to open-ended questions, interviews and focus group data. KW read interview transcripts several times to compare between and within transcripts,^31^ ^32^ and ensure that account was taken of the wider context of participants’ accounts. ‘Deviant’ cases helped to inform the analysis and are presented below to assist transparency in describing our interpretations of the data.^27^ KW reviewed and discussed the developing coding framework during regular meetings with LF and BY. KW analysed quantitative questionnaire data using simple descriptive statistics and χ^2^ test.

Our approach to synthesising the qualitative data, quantitative data and ethical theory^24^ was pragmatic, and drew on the constant comparative method.^34–36^ For example, KW cross referenced qualitative themes with subject-related statistical output from the questionnaire analysis in order to present overall themes on a given topic. No one type of data was given precedence.^24^ ^37^ Where qualitative and quantitative findings on an issue did not corroborate or when there was divergence between accounts on the same key issue, data sets were further explored^37^ or further interviews were conducted in order to assist understanding and interpretation. Findings were then considered and explored in the light of bioethical principles, including voluntariness, autonomy, non-maleficence and justice^5^ ^38^ to help draw practice-orientated conclusions that were ethically defensible. This included considering the data in relation to the circumstances which impact on experiences of trial recruitment in this setting, and reflecting on how key theories and principles could inform the analysis.^38^ Regular meetings were held between KW, LF and BY to review the developing analysis. For each key theme we present overarching findings from qualitative and quantitative data sets, unless the issue was evident in only one type of data. We use quotations to illustrate our findings; brackets […] indicate that text has been removed for brevity.

## Results

### Sample

**Figure 1:**
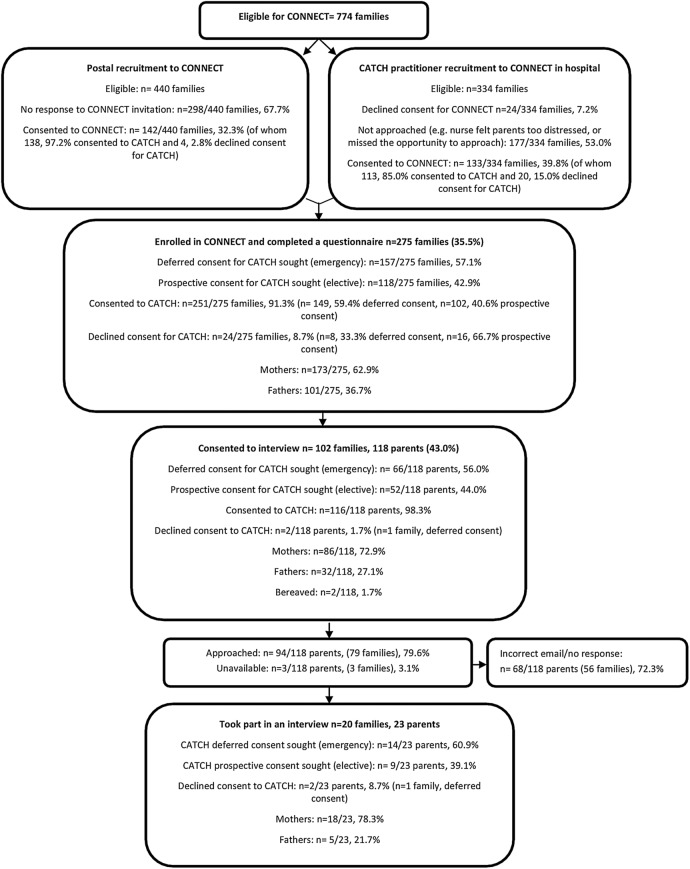
CONNECT parent recruitment process and sample characteristics. CATCH, CATheter infections in CHildren.

Two of 14 CATCH hospital sites did not participate in CONNECT due to research governance delays. A total of 774 families were eligible for inclusion in CONNECT. As shown in figure 1, this included parents who had declined CATCH as well as those who consented. Of these, 440 parents had indicated interest in future research via the CATCH consent form before CONNECT began (postal recruitment to CONNECT) and 334 had been recruited to CATCH after CONNECT began (practitioner recruitment to CONNECT).

As shown in figure 1, 275/774 (35.5%) eligible parents from the 12 CATCH sites enrolled in CONNECT and completed a questionnaire. This included 142/275 (51.6%) parents recruited via postal invitation and 133/275 (48.4%) parents recruited by a CATCH practitioner in hospital, comprising 173 mothers (62.9%), 101 fathers (36.7%) and one guardian (0.4%). Just over half (157/275 (57.1%)) were emergency admissions and had been approached for deferred consent, while 118/275 (42.9%) were elective admissions and had been approached for prospective consent. Of the 275 parents in CONNECT, 24 (8.7%) had refused consent for CATCH, 8 parents were in the emergency arm and 16 were in the elective arm. Eight of 275 (3%) were parents of children who had died in the course of their treatment, of whom all had provided deferred consent. Parents approached for deferred consent (emergency arm n=149/275, 54.2%) were significantly (p=0.01) more likely to provide consent for CATCH than those approached for prospective consent (elective arm n=102/275, 37.1%).

Of the 118/275 (43.0%) parents who agreed to be approached for interview, 94 (79.6%) were purposively selected and invited (see figure 1). Of these, 68/94 (72.3%) did not respond or the email addresses were incorrect, while three parents were unavailable for interview due to their child's illness. A total of 20 families (18 mothers and 5 fathers) were interviewed by telephone (n=16 parents) or face-to-face in their homes (7 parents). These parents were drawn from six (n=6/12, 50%) CATCH sites; 14 had children who had been admitted as an emergency and approached for deferred consent. The children of two parents had died during their hospital admission and one of these parents had declined consent for CATCH. Interviews with parents took between 40 and 90 min, focus groups discussion with practitioners took approximately 90 min.

**Figure 2:**
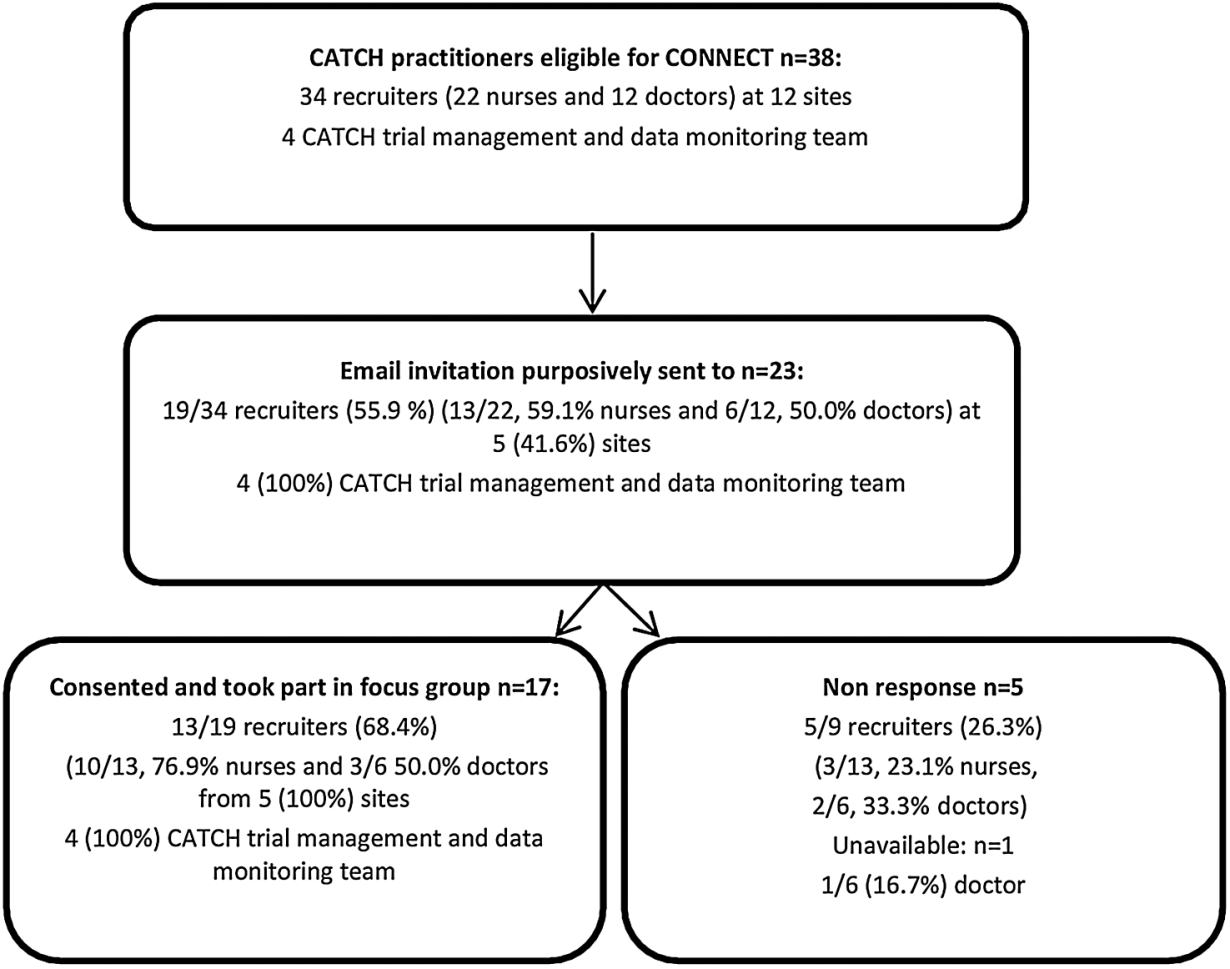
CONNECT practitioner recruitment process and sample characteristics. CATCH, CATheter infections in Children.

As shown in figure 2, KW conducted five focus groups with 17 of 23 (73.9%) invited practitioners. Four focus groups involved CATCH recruiting practitioners, including 10/13 (76.9%) invited nurses and 3/6 (50.0%) invited consultant grade doctors from five preselected trial sites. All but one CATCH practitioner was involved in the clinical care of children. One further focus group involved four (100%) members of the CATCH trial management and monitoring team based in the MC CTU.

### Parents’ understanding of the CATCH consent sequence and initial concerns

During the early stages of interviews, KW explored parents’ recollection of the CATCH recruitment and consent process, including the time point at which their child's catheter was inserted in relation to the time point at which a doctor or nurse had approached them to discuss CATCH and seek consent.

Most parents accurately described the sequence of events, including the timing of insertion of the trial catheter in relation to the timing of consent for CATCH: “I know that the form was signed the day before the operation” (P15, mother, elective group, recovered). However, three parents of children who had been admitted to paediatric intensive care could not recall the order of catheter insertion and being approached to provide consent for CATCH: “I can’t honestly remember which came first” (P17, female, emergency group, recovered). Despite not being able to recall that consent had been deferred for their child's participation in CATCH, two parents responded positively to KW's explanation of deferred consent. These parents described how the emergency treatment of a child should be prioritised over research consent procedures: “I would rather that action be taken first to consider the person, you know, who is ill, to consider their wellbeing rather than fanny around with paperwork” (P11, mother, emergency group, recovered). In contrast, the third parent, a bereaved mother, was shocked that her child had been entered into CATCH without her prior consent and questioned what would have happened if she had declined consent for CATCH: “Um, it’s a bit of a shock that, that he'd kind of been entered into a trial […] I don’t know, what they would have done if we’d have said no?” (P20, mother, emergency group, bereaved).

Among parents who recalled their initial responses to deferred consent in the emergency setting, some seemed unperturbed by the consent arrangements “I wasn't worried by that at all” (P8, mother, emergency group, recovered). Approximately half of parents made remarks that implied they were surprised to learn that it was possible for consent seeking to be postponed in this way: “I didn't know it existed to be honest” (P14, mother, elective group, recovered), or described how they had been initially “a bit shocked that they'd put […] a central line into [child name] that could have been one or one of three different types without us knowing [laughs]” (P12, mother, emergency group, recovered). One mother described an initial sense of dismay at the use of deferred consent: “I wasn't very happy about it, initially, that it had been done without, without asking our consent first” (P12, mother, emergency group, recovered). We also explored the views of parents who had experienced prospective consent within CATCH about the wider application of deferred consent in children's emergency trials. A similar proportion (approximately half) to the emergency group of parents responded negatively, explaining how they would have been “annoyed” (P4, father, elective group, recovered) or uneasy if their consent had not been sought prospectively: “you're just not asking first […] it's really hard and it's a really tricky one” (P14, mother, elective group, recovered). One mother pointed to how deferred consent compromises a parent's right to make decision about their child's participation in research: “I'm not sure whether I completely agree with it […] because it's effectively already been done, hasn't it really? So I think it takes away your power as a parent to make that decision” (P16, mother, elective, recovered). However, as we shall discuss later, these parents’ response to deferred consent changed over the period of the CATCH consent discussion or the interview discussion of deferred consent, and none of the practitioners interviewed described any of the parents having initial negative responses to deferred consent.

### Practitioners’ views about deferred consent before experiencing it in CATCH

At the beginning of focus group discussions, practitioners explained that prior to CATCH they did not have any previous experience of deferred consent. Almost all nurses were initially apprehensive about implementing deferred consent. Nurses were particularly concerned about how parents would respond to deferred consent: “The first time I did approach a parent I remember feeling really nervous about their reaction” (P7, female nurse, focus group 2). In contrast, three consultant grade doctors and one nurse described how they welcomed deferred consent without reservation “I thought fantastic” (P11, female doctor, focus group 4) as it enabled trials to be conducted in emergency settings. For one doctor, the sequencing of communication in deferred consent was not dissimilar to that in clinical care: “We'll frequently tell people afterwards what we've done to their child so that sort of telling them afterwards […] so the concept is quite… It's not alien” (P11, female doctor, focus group 4).

### Parental acceptance of deferred consent hinged on practitioner explanation and perceived safety

During interviews, parents described how practitioners’ explanations about why a deferred consent approach is used in the emergency settings had helped to dispel their initial shock or concern about why their prior consent had not been sought. Returning to one of the parents in the emergency arm, who was initially shocked about the deferred consent process: “I was really surprised at first and I wasn't very happy about it initially, that it had been done without, without asking our consent first but once they'd explained why […] and I don't really think on reflection that there was any way […] to do it […] all that matters to you is your, your child, but it's important that the research happens” (P12, mother, emergency group, recovered).

The nature of the investigational ‘treatments’ being compared also appeared to influence parents’ views on the acceptability of deferred consent. CATCH was a medical device trial involving three catheters that were already in routine use in hospitals across the UK, so nothing novel was being administered to children as part of the trial. Moreover, inserting a CVS is a routine part of emergency treatment and there was little change in a child's overall care as a result of being randomised to the trial. These factors were important to parents and appeared to influence their views on when it was appropriate to use deferred consent: “Because there wasn't any harm to the patients, and it was an emergency situation they had to have a catheter put in because it was life or death, then it really, you know, didn't, didn't make much difference whether they were doing it or not” (P13, mother, emergency group, recovered).

### Acceptance of deferred consent in CATCH and support for its use in other trials

The CONNECT questionnaires, which parents completed after being invited to enter their child into CATCH, included a series of statements to assess parental satisfaction with the CATCH consent process, as well as their sense of whether or not they felt their decision-making was voluntary. As table 2 shows, there were no statistically significant differences (p>0.05) between groups (elective or emergency) for any of the responses to statements posed to parents in the questionnaire. Despite some parents voicing initial concerns about deferred consent during interviews, no parents in the elective or emergency arm expressed dissatisfaction with the consent process in their questionnaire responses (statement 1). A slightly higher proportion of parents in the elective arm (n=68/117, 58%) strongly agreed with statement 1, indicating they were satisfied with the consent process for CATCH, compared to those in the emergency arm who experienced deferred consent (n=74/156, 47%; p=0.09). A slightly higher proportion of parents in the emergency arm who experienced deferred consent (n=105/155, 67%) strongly agreed with statement 3: ‘I made this decision’ compared to parents in the elective group (n=70/118, 59%; p=0.17) where consent was sought prospectively.

Interview findings broadly support the questionnaire findings. Despite some parents indicating in their interviews that they had initially been ‘unhappy’ or ‘surprised’ when approached for deferred consent; after the doctor or nurse had explained the reason why consent had been deferred in CATCH, parents described how they were ‘quite happy’ (P13, mother, emergency, recovered) with the consent process. No parents described a sense of lasting upset or anger that their child had been randomised to CATCH; rather parents went on to speak about their support for the use of deferred consent in CATCH and other paediatric emergency trials provided that these did not involve a new investigatory treatment or a change in clinical care: “As long as it doesn't affect the care that your child's receiving then I don't see that there's a problem” (P8, mother, emergency, recovered). Support for deferred consent was regardless of whether or not parents had first-hand experience of the approach. Parents in the elective arm who had expressed initial anger or concern about the method also went on to speak of their support for deferred consent when the reasons for using this method were explained by the researcher: “if it was emergency medicine, yes I would be happy with that [deferred consent]” (P16, mother, elective, recovered).

We further explored this acceptance of deferred consent in CATCH by asking parents about their views on the potential use of deferred consent in other emergency trials, which might not always involve investigational treatments already used as a routine part of emergency treatments. Many parents were concerned about the use of deferred consent in trials involving ‘new’ drug interventions that were not already used in clinical care or trials that involved a potentially significant change in clinical practice: “If it was some kind of test where they were trying something totally radical […] like a real departure between what treatment she would have received and what treatment she did receive, then I guess I'd have… had a… I would have questions” (P22, father, emergency group, recovered).

In the latter part of focus group discussions, practitioners reflected on their experience of the CATCH trial, often describing how their initial apprehensions about parents’ responses to deferred consent were not realised. They spoke of how parents were: “Very receptive” (P14, male nurse, focus group 1) and responded positively to the deferred consent discussion: “The majority of the time they were very, very happy” (P6, male nurse, focus group 2). Practitioners referred to a small number of parents who had declined deferred consent, attributing these declines to practitioners not having approached parents “at the right time” (P8, female doctor, group 3) or some parents not wishing their child to take part in any ‘research’ rather than dissatisfaction with deferred consent in CATCH: “It's [research] okay per se but not on my child” (P17, female nurse, focus group 1).

### Conflicting views on deferred consent for blood samples

The CATCH primary outcome (time to first blood stream infection) was measured by a blood sample (0.5 mL) taken from all catheter lumens (total 1.0–1.5 mL, depending on whether the catheter had two or three lumens) if there was a clinical indication of infection (see table 1). These samples were required as part of standard, good clinical practice and were not an additional requirement of the study. The CATCH protocol, stated that for emergency admissions, blood samples could be taken prior to seeking consent: “Because blood sampling from all catheter lumens is the standard of good practice used in the trial, this sampling method should be used for patients who have not yet been approached for deferred consent.”^39^ However, the protocol required a small amount of additional blood to be taken (approximately 0.5 mL) to test for bacterial DNA (called PCR testing). This was required for a secondary, composite measure of blood-stream infection. This test was additional to standard care.

All doctors and two nurses interviewed described how they were not concerned about deferring consent for a blood sample: “It's just a bit of blood” (P11, female doctor, focus group 4), remarking that the blood sample was small and insignificant in the context of the wider emergency interventions and the blood sampling that a child's clinical management would require. In contrast, most nurses interviewed were concerned that taking blood samples for PCR testing for research rather than clinical need, without prior informed consent, would compromise a trusting parent–practitioner relationship. Some nurses also explained how CATCH required them to take a significantly larger amount of blood for blood cultures than was normally taken within their unit to establish if there was a blood stream infection (0.5 mL from all lumens compared to 1 mL from one lumen, plus 0.5 mL for the PCR test): “it was such a huge difference between our norm… we said that we weren't happy to do it” (P16, female nurse, focus group 1). They also reported concerns among families that blood samples might contribute to the need for blood transfusions in small neonates: “I've had a parent ask me, “If you weren't taking these blood samples, would they need a blood transfusion?” I said, “I honestly can't say yes or no.” (P16, female nurse, focus group 1). Nurses in three of the four sites that participated in the practitioner focus groups reported refusing to take blood samples until after consent had been obtained. No doctors described refusing to take such samples. During focus group discussions, some practitioners reflected on how it was nurses, rather than parents, who became ‘upset’ about deferred consent for additional blood samples, particularly when consent was declined and a child's blood samples (taken prior to consent) had to be destroyed.

A few parents recalled that prospective consent had been sought for blood sampling as part of CATCH. However, many parents interviewed who had experienced deferred consent were uncertain about the order of consent and blood samples. Three parents were unsure about whether additional blood samples had been taken as part of the trial although the need for ‘a little bit of extra blood’ had been mentioned in the CATCH patient information leaflet. This was an important issue, as blood sampling seemed to be a factor in these parents’ decisions to provide deferred consent. As one bereaved parent described: “We may have wanted a bit more information and things, but, um, or, you know, taking blood tests or stuff like that. But this one (study) it didn't seem to ask any of that, so yeah we were happy” (P20, mother, emergency, bereaved). When parents were asked about their views on the acceptability of deferred consent for blood samples, none spoke of being upset or unhappy that blood had or could be taken for CATCH without their prior consent: “because the bloods are not going to cause any harm to him” (P4, father, elective, recovered). One mother mentioned that “it would have been nice if you were asked” (P15, mother, elective, recovered), although she was not unhappy with the deferred consent approach, in general. Another parent spoke of her fear that her son was ‘bleeding out’, which had prompted her to ask a nurse about the amount of blood that would be taken for CATCH. However, the mother pointed to how the nurse's explanation had allayed her initial concerns, adding that she didn't have “any major concerns about […] you know, having any blood taken beforehand” (P12, mother, emergency, recovered).

From the focus groups in the trial management team it was clear that the team had not anticipated nurses’ concerns. Trial managers added that such non-adherence to the protocol “wasn't a huge problem” (P3, female, CATCH trial management team, focus group 5) for this trial. However, they spoke of how, in other emergency trials, staff refusal to take additional blood samples before seeking deferred consent could invalidate a trial's results if this introduced bias or led to missing data.

### Why deferring consent is better than seeking prospective consent—but it's important to ‘pick the right time’

During interviews, parents reflected on how they would be unable to concentrate on explanations about a trial or make an informed decision if practitioners had attempted to seek their consent when their child had first been admitted to intensive care: “In the first half day, I don't think I would have taken on board anything anyway. I can't really remember lots […] I may well have agreed to do the trial, but I wouldn't have concentrated on it as much as I did later on” (P12, mother, emergency group, recovered). One parent described how he would “have said no” to any trial “if they'd have asked us as he was being admitted” (P4, father, elective group, recovered) because his child was critically ill and it was not an appropriate time to discuss research. Rather than seeking consent prospectively in an emergency situation, parents were clear that “it’s better that they ask me later” (P20, mother, emergency group, bereaved) and “when everything has settled down” (P1, mother, emergency group, recovered). Parents described how the nurse had “picked the right time to come” (P13, mother, emergency group, recovered) to discuss CATCH, which was after the immediate emergency situation had passed: “they did wait until it was all quite calm” (P20, mother, emergency group, bereaved). From the perspective of practitioners deferring consent enabled them to approach parents “at a time when we feel that they are able to absorb the information” (P5, male nurse, focus group 2). Questionnaire findings complemented qualitative interview findings in indicating that the timing had been appropriate from the parents’ perspective. The majority of parents in both trial arms either strongly agreed (139/275, 51.0% ) or agreed (115/275,41.8%) with statement 2 that they had had sufficient time to think about whether or not to consent for their child to take part in CATCH (see table 2). The majority of parents also strongly agreed (161/275, 58.5%) or agreed (108/275, 39.3%) that they had enough opportunity for questions about CATCH.

However, some parents’ interview accounts of their CATCH recruitment experience in the elective and emergency trial arms suggested insensitive and untimely approaches had been made: “When the nurse came back the following day unfortunately that really wasn't a good time because that was at the point at which they’d told us that they thought that he was brain dead” (P12, mother, emergency group, recovered). Parents had been approached “right in the middle of all the massive important stuff” (P4, father, elective group, recovered), which they reported had compromised their ability to ‘digest’ trial information and make an informed decision. One family described how a nurse had tried to give them ‘loads of information’ when their child had just been lifted off “the ambulance bed onto the intensive care bed” (P1, mother, emergency group, recovered), an experience which they said had led them to immediately decline CATCH. Parents’ accounts of such events suggest that in some cases practitioners mistimed the recruitment discussion. Parents described how the trial information would have been “more digestible” (P4, father, elective group, recovered) at a different point of time. One family reflected on how “If she came round at a better time, then I think we would have approved it” (P1, mother, emergency group, recovered), indicating the importance of ensuring that the timing of discussions about research did not interfere with a parent's need at the height of the critical situation for their child to be their only focus.

### Deferred consent for CATCH was an ‘easy decision’ but not always an informed one

As the trial catheter had already been inserted, parents could take their time to consider deferred consent. However, many described how they provided deferred consent quickly: “We were quite positive about it and quite receptive to it [CATCH], so, you know, within the conversation I don’t think it was more than kind of ten minutes we were happy to sign and say yes” (P20, mother, emergency group, bereaved); as “It was quite an easy decision” (P23, mother, emergency group, recovered). Some parents drew comparisons between the decision about CATCH and clinical-care decisions: “It was one of the easiest ones I made in those forty-eight hours” (P9, mother, emergency group, recovered). Nurses also spoke of the emergency context and how parents’ deferred consent decision seemed relatively insignificant in the context of such a traumatic situation. Although some parents interviewed had a clear understanding of the trial device: “he arrived they put the central line […] to monitor his blood pressure and get all of the drugs and things into him” (P12, mother, emergency group), others described how they had thought the term CVC referred to a urinary catheter: “I thought the catheter was his wee thing” (P2 father, emergency, recovered). This parent went on to explain how doctors and nurses treating his child referred to CVCs as ‘lines’ rather than ‘catheters’, which was different to the description provided in the trial information leaflet and may have confused parents. As we have described above, some parents did not understand that CATCH involved taking blood samples from children despite this being included in the patient information sheet, and some parents also ‘misunderstood’ the sequence of administering the trial interventions. These findings suggest that although parents felt the CATCH deferred consent decision was relatively easy to make, it was not always a well informed decision.

### The moral and emotional burden of seeking consent after a child has died

Ethics committee advice for CATCH was that: “in circumstances where children die before consent has been obtained the patient information sheet should be given as soon as possible, but timing for the approach for consent could be decided by the clinician.” Practitioners were, therefore, required to contact bereaved parents to seek deferred consent.

During focus group discussions, nurses and doctors described their apprehensions about approaching bereaved parents for deferred consent: “To ask them later, when the child is dead, is much more difficult” (P8, female doctor, focus group 3) as they “didn't want to burden them” (P11, female doctor, focus group 4). Practitioners spoke of their dismay at having to “chase” bereaved parents “at the most stressful, awful time in their lives” for a consent decision. Nurses described how it was often a senior member of the team who contacted parents: “he wasn't particularly happy about doing that either” (P7, female nurse, focus group 2). Two sites made the decision not to “go chasing via phone calls; we didn't think that was appropriate” (P7, female nurse, focus group 2). From the perspective of practitioners, a generic letter or phone call about research in the aftermath of a child's death lacked compassion for the devastation that parents will feel at this time. Nurses at one site described how they personalised the letter to acknowledge the relationship they had with parents: “We also reworded the letter to send it out, because for some you'd already approached so therefore you needed that, you know, we've met you before. So it made sense to have that kind of more personal” (P17, female nurse, focus group 1). In some cases there was a consultant led decision not to make contact, based on their prior relationship with the family: “There were a couple where I didn't send them at all […] it depended a bit what type of relationship there was prior, whether I felt err they would be burdened or not” (P11, female doctor, group 4).

Practitioners described seeking consent as “incredibly difficult…” and admitted to fears that such conversations would cause parents “additional upset and stress” (P9, female nurse, focus group 3). One nurse described how: “Some parents did actually say to me, why are you asking me this? And then I just feel like the worst person in the world” (P9, female nurse, focus group 3). However, the majority of doctors and nurses described their relief and surprise that there had not been any negative repercussions from sending the letter to request deferred consent from bereaved parents: “I'm shocked that we haven't had any complaint letters back” (P16, female nurse, focus group 1). While practitioners spoke of how there was no ‘perfect solution’ to approaching parents for consent after their child has died, some commented that consultation with bereaved parents would help future trial teams to enhance the way they approach bereaved parents for consent: “That has to come from the experiences of the families, to sort of say what is acceptable at that time” (P7, female nurse, focus group 2).

The one bereaved parent who we interviewed had a poor recollection of the CATCH consent discussion and the sequence of events, but remarked on her sense that staff had “asked at the right time” as research was “last thing on my mind when, when we went in in the emergency situation” (P20, mother, emergency, bereaved). This mother spoke of how using her child's data in the trial was “something positive… to help others or to, to further the, the research”, although she described how establishing the best time to approach bereaved parents was “a tough one” suggesting that nurses would be “the best people to, to know when would be an appropriate time.”

### Child assent

Ethical guidelines require that children's assent is sought for medical research if they are competent to do so.^40^ ^41^ Owing to the emergency situation, the CATCH protocol stated that assent was to be sought from children “as soon as their condition allows.”^39^ Of the 1485 children participating in CATCH, 274 (18.5%) were of an age (>5 years) typically considered suitable to allow meaningful engagement in assent discussions (95 of whom were in the elective arm), although only three forms documenting children's assent were received across both the elective and emergency arms of the trial. When we explored this during the practitioners focus group discussions, they pointed to the young age profile of CATCH participants and the condition of children who may have been ventilated or were “a bit drowsy” (P11, female doctor, focus group 4) as explanations. Practitioners also described how seeking child assent was often impossible or inappropriate due to time constraints or because children had “developmental delays” (P9, female nurse, focus group 3).

## Discussion

### Main findings

We believe this is the first UK study to explore the views and acceptability of deferred consent among parents and practitioners with first-hand experience of such a consent process. In line with our wider CONNECT findings, CATCH practitioners described how they were initially apprehensive about using deferred consent arising from their lack of previous experience with the method.^22^ Deferred consent seemed to initially perturb some parents, who spoke of their momentary shock or anger when they first found out that their child had been entered into research without their prior knowledge and consent.^42^ Many parents were surprised that the normal process of research information disclosure as part of an informed consent process could be changed and postponed in this way,^40^ ^43^ while a few suggested that their right as a parent to make an autonomous decision about research had or could have been taken away.^5^ ^44^ ^45^ Practitioners did not describe these initial negative reactions from parents, suggesting that parents did not voice these concerns during recruitment discussions.^46^ However, our interviews, focus group discussions and survey findings indicate that parents and practitioners’ initial concerns about deferred consent were short lived. Hearing practitioners explain why deferred consent is needed to enable research to be conducted in time limited emergency situations appeared to dispel parents’ concerns. No parents in our study described a sense of lasting upset or anger that their child had been randomised to CATCH without their prior consent. However, a few parents were unhappy about consent—whether it was deferred or prospective—having been sought at a time they felt was inappropriate. Analysis of questionnaires completed by parents after their CATCH consent discussion indicated that their decisions had been made voluntarily, while in the latter part of interviews, parents described their support for deferred consent in CATCH and for other emergency trials. This supports wider CONNECT study findings showing that parents’ initial concerns can change when the reasons for deferring consent are explained,^22^ and indicates the importance of practitioner communication^47^ in deferred consent discussions.

Inserting a CVS is a routine part of emergency treatment; the CATCH trial arms were very similar and there was little change in a child's care as a result of being randomised to the trial. Practitioner's explanations of these factors and how the trial did not pose any additional risks to child safety appeared to positively influence parents’ views on those situations in which it seemed appropriate to use deferred consent. This qualitative finding supports our wider research that has shown that parents support deferred consent in order to enable research to progress in children's emergency medicine, so long as the child's safety is not compromised.^4^ However, it raises ethical concerns about the use of deferred consent for future trials where the trial intervention is not already used in routine clinical practice, involves a change in clinical practice, requires taking more blood, or where safety cannot be so readily assured. As parents of critically ill children are sometimes prepared to accept higher risks if there is a chance that their child's illness could be cured or improved,^48^ there is a need for further research to explore the acceptability of deferred consent for trials that, while involving higher risks, provide treatment options for critically ill children that might not otherwise be available. Although nurses’ refusal to defer consent for blood samples did not greatly impact on CATCH findings,^49^ such refusal may have implications for other critical care trials, either burdening families if they are approached for prospective consent at an unsuitable time, invalidating trial results, introducing bias or resulting in missing data.^50^ ^51^

### Strengths and weaknesses

This was a mixed method substudy involving 12 of 14 hospitals that took part in the final year of CATCH. Comparison of the consent rates for CATCH between the CONNECT subsample and the wider sample of parents approached to participate in the trial was not possible due to incomplete CATCH screening data. We strengthened our qualitative sampling by conducting interviews until no new relevant knowledge was obtained from new participants (data saturation).^27^ One person (KW) conducted all interviews. A research diary was kept to record field notes and assist self-reflection and transparency in the research process.^27^ ^29^ We maximised diversity within our qualitative sample by selecting for interview both mothers and fathers; those who had consented and those who had declined consent for CATCH; as well as parents who did and those who did not have experience of deferred consent. However, opportunities to purposively sample parents who declined consent and bereaved parents were limited due to high consent rates and low death rates in the trial. This limits our understanding of the experiences of these groups and the recommendations we can make to inform how deferred consent should be sought when a child has died. As parents recruited via postal invitation were limited to those who indicated on their CATCH consent form that they wished to take part in further research, our sample is, therefore, more likely to comprise parents who were interested in research. A higher proportion of nurses than doctors took part in our study; this reflected the trial nurse to doctor ratio within participating sites. Our insight into consent discussions was limited as we relied on parents’ and practitioners’ recollections. However, our findings were strengthened by accessing the perspectives of parents and practitioners, and by the use of mixed methods, which enabled us to gain a rounded, multiperspective understanding of their views and experiences of deferred consent.^26^ Attempts were made to include children in our study; however, child's assent was rarely sought in CATCH, which practitioners attributed to there being insufficient time to seek assent or that the children were too young or still sedated for discussions prior to discharge. These issues pose a significant barrier to children's involvement in decisions about their participation in future paediatric critical care trials.

### Consideration of findings in relation to other studies

Our findings add to evidence from a few studies^4^ ^18^ ^19^ that have explored the acceptability of deferred consent among parents without direct experience of the method and indicate general support for the use of deferred consent in children's critical care trials. As other studies have shown, clearly communicating and helping patients to understand trial information is a challenge for practitioners in any trial^52^ ^53^ and parents in the non-emergency setting may sign consent forms and consider themselves informed without an adequate understanding of the research or how it impacts on their family.^46^ ^54^ ^55^ Our study provides new evidence that deferring consent to a time point after their child's condition had stabilised enabled most parents to have a sense that they could consider trial information, and that parents felt such timing was more appropriate than seeking consent at an earlier and more critical time point. Practitioners also believed such timing assisted informed decision-making. Our survey findings indicate that parents felt they understood the information they received about the CATCH trial, and were able to make voluntary and informed decisions about the use of their child's data and for their continued participation in the trial.^5^ ^56^ However, qualitative insight gained through interviews indicated that some parents were unaware that they had experienced deferred consent until they took part in a CONNECT interview. Others were confused about the nature of the trial device or blood samples, which had the potential to influence their decision-making.^46^ While we do not know how well practitioners explained these issues, the CATCH information sheets provided to all parents described the nature of the intervention, sequence of catheter insertion and reasons why consent had been deferred, including how additional blood would be taken. These information sheets may not have been read or understood by a few parents.^57^ ^58^ It is very likely that the emergency situation (involving the death of a child, in one instance) impacted on these parents’ capacity to absorb and understand information,^1^ ^59^ even when consent was deferred until after the critical situation had passed.

The similarity in initial responses to deferred consent among parents who did and did not have direct experience of the method suggests that ‘hypothetical’ pretrial studies involving parents without experience of the method may be useful to inform future trials in this setting. However, only interviews with experienced parents provided insight into issues, such as parents perceptions about the sequence of administering the trial interventions and blood samples, suggesting that researchers conducting trial feasibility or pilot studies should consider conducting qualitative research involving those with direct experience of recruitment to critical care trials to optimise approaches to recruitment, consent or the conduct of the trial.^60^

### Implications for practitioners when seeking deferred consent

Our study provides evidence that can be used by practitioners, and patient and public involvement partners involved in the design, ethical approval and conduct of children's critical care trials to improve how consent is sought in the emergency setting. Where deferred consent is being considered for a potentially challenging trial (eg, trials involving change in clinical practice such as a new or novel intervention), the views of parents, children and practitioners should be systematically sought through substantive research at the pretrial (eg, feasibility or pilot) stage to inform the trial design, recruitment and approach to consent. Unlike the ‘exception from informed consent (EFIC)’ approach used in the USA,^61^ which requires community consultation for all emergency research, we suggest that substantive research at the pretrial stage is only necessary when deferred consent is proposed for trials where a child's safety cannot be readily assured. Ideally, samples for such feasibility studies should include a diverse group of parents and their children (where applicable) who experience the health condition being investigated by the trial or have experienced the processes involved.

When seeking deferred consent, it is important that practitioners assess the timing of a recruitment discussion through consultation with colleagues. This includes explaining what deferred consent is and why it is being used. To assist parental understanding and decision-making,^22^ aspects of trial information should be clarified, such as the nature of trial interventions, any potential risks as a result of being included in the trial, whether and how the child's care has changed, and whether interventions are used in standard clinical practice. As practitioners inexperienced in deferred consent may be apprehensive about discussing this method with parents,^22^ and parents may react negatively or struggle to voice their concerns in recruitment discussions with practitioners,^46^ CONNECT findings should be fed into practitioner training for future critical care trials that use deferred consent. Practitioners with first-hand experience of deferred consent should be involved in the design and ethical review of future trials of emergency treatments in children.^22^

### Unanswered questions and future research

Excluding data on children who die will bias trial findings and previous research has shown that bereaved parents wish to be informed about their child's participation in a trial although a minority do oppose such disclosure.^19^ ^62^ Our findings indicate the emotional and moral burden practitioners experienced when approaching bereaved parents for deferred consent. Further research is required involving bereaved parents to inform how and if consent should be sought for a trial when a child dies. Such research should also explore bereaved parents’ views on the inclusion of children's data in a trial when practitioners have made attempts to seek deferred consent but parents have not responded.

Future research would benefit from recording deferred consent discussions^36^ ^46^ to inform future training by providing insight into how practitioners explain deferred consent to parents and to identify whether parents voice their initial concerns. Further research is required to explore whether seeking the child's assent is challenging for practitioners in other emergency research settings and whether there are alternative ways of involving older children in decisions about their participation in critical care trials, such as contacting them at their local hospital (if applicable), at home or through their general practitioner when they have recovered. Future research involving parents and practitioners at the pretrial stage may help to identify issues that have the potential to negatively impact on consent and trial recruitment, and how it is experienced^46^ in order to tailor protocol, patient information^57^ and practitioner training to the needs of parents before the trial begins.^60^ Qualitative research embedded within other trial types, such as trials of medicinal products and interventions not used in standard clinical care, would enhance our understanding of the acceptability of deferred consent to inform future practice.
